# Structural basis of the interaction between the putative adhesion-involved and iron-regulated FrpD and FrpC proteins of *Neisseria meningitidis*

**DOI:** 10.1038/srep40408

**Published:** 2017-01-13

**Authors:** Ekaterina Sviridova, Pavlina Rezacova, Alexey Bondar, Vaclav Veverka, Petr Novak, Gundolf Schenk, Dmitri I. Svergun, Ivana Kuta Smatanova, Ladislav Bumba

**Affiliations:** 1 Faculty of Science, University of South Bohemia Ceske Budejovice, Branisovska 1760, 37005 Ceske Budejovice, Czech Republic; 2 Center for Nanobiology and Structural Biology, Institute of Microbiology, Czech Academy of Sciences, Zamek 136, 37333 Nove Hrady, Czech Republic; 3 Institute of Organic Chemistry and Biochemistry, Czech Academy of Sciences, Flemingovo nam. 2, 16610 Prague, Czech Republic; 4 Institute of Molecular Genetics, Czech Academy of Sciences, Flemingovo nam. 2, 16610 Prague, Czech Republic; 5 Institute of Microbiology, Czech Academy of Sciences, Videnska 1083, 14220 Prague, Czech Republic; 6 EMBL Hamburg Outstation, c/o DESY, Notkestrasse 85, D-22603 Hamburg, Germany

## Abstract

The iron-regulated protein FrpD from *Neisseria meningitidis* is an outer membrane lipoprotein that interacts with very high affinity (*K*_*d*_ ~ 0.2 nM) with the N-terminal domain of FrpC, a Type I-secreted protein from the Repeat in ToXin (RTX) protein family. In the presence of Ca^2+^, FrpC undergoes Ca^2+^ -dependent protein *trans*-splicing that includes an autocatalytic cleavage of the Asp_414_-Pro_415_ peptide bond and formation of an Asp_414_-Lys isopeptide bond. Here, we report the high-resolution structure of FrpD and describe the structure-function relationships underlying the interaction between FrpD and FrpC_1-414_. We identified FrpD residues involved in FrpC_1-414_ binding, which enabled localization of FrpD within the low-resolution SAXS model of the FrpD-FrpC_1-414_ complex. Moreover, the *trans*-splicing activity of FrpC resulted in covalent linkage of the FrpC_1-414_ fragment to plasma membrane proteins of epithelial cells *in vitro*, suggesting that formation of the FrpD-FrpC_1-414_ complex may be involved in the interaction of meningococci with the host cell surface.

The Gram-negative bacterium *Neisseria meningitidis* is a strictly human commensal that colonizes the nasopharynx of approximately 10% of healthy individuals. Occasionally, hyperinvasive meningococcal clones spread in the population and cause endemic cases, local outbreaks, or even epidemics of invasive meningococcal disease with high morbidity and mortality rates^1^,^2^,^3^,^4^. *N. meningitidis* expresses a range of virulence factors, including capsular polysaccharide, lipopolysaccharide, and several types of partially redundant surface-exposed adhesive proteins that are involved in colonization of host tissues and bacterial survival on mucosal surfaces^5^. In addition to the major adhesins, such as type IV pili and the opacity proteins (Opa), several minor adhesins (NhhA, App, NadA) support bacterial colonization and invasion of the mucosal barriers^6^,^7^,^8^,^9^.

Iron starvation is a key signal controlling the expression of virulence factors. The iron response in meningococci is mediated by the ferric uptake regulator (Fur) protein, which binds to a consensus promoter sequence (Fur box) and transcriptionally regulates the expression of many virulence factors and iron-regulated genes^10^. Of these, *frpD* and *frpC*, are encoded in the *frpDC* operon and are overexpressed by *N. meningitidis* grown under iron-limited conditions^11^,^12^.

The *frpC* gene product, FrpC, is a 1829-residue protein and its variously truncated or extended variants are secreted into the extracellular environment during the early stages of meningococcal infection^13^. FrpC belongs to the family of Repeat in ToXin (RTX) proteins, which is characterized by the presence of successive blocks of tandemly repeated C-terminal RTX nonapeptides with the consensus motif GGxGxDxxx^14^. These nonapeptides bind Ca^2+^ ions, and their sequential folding promotes secretion of RTX substrates from the Ca^2+^-free bacterial cytosol into the Ca^2+^-rich extracellular milieu through a dedicated Type I secretion system (T1SS)^15^. Even though many RTX proteins serve as virulence factors for Gram-negative bacteria, FrpC does not seem to exert any cytotoxic activity, and its biological function remains unknown^16^,^17^. However, FrpC undergoes an autocatalytic and highly specific processing, which closely resembles a process known as “protein *trans*-splicing” (Fig. 1). This process includes Ca^2+^-induced autocatalytic cleavage of the Asp_414_-Pro_415_ peptide bond and a subsequent covalent linkage of the released carboxyl group of Asp_414_ of the FrpC_1-414_ fragment to an adjacent ε-amino group of another lysine residue through an isopeptide bond^18^. The *trans*-splicing activity of FrpC is mediated by a self-processing module (SPM), an adjacent segment of 177 residues (415–591), the primary amino acid sequence of which appears to be well-conserved in many RTX proteins from Gram-negative pathogens^19^. The cleavage activity of SPM is closely associated with a Ca^2+^-induced conformational switch from an intrinsically unstructured state to the folded state^20^. This can be efficiently dissociated from the cross-linking activity of SPM in the presence of nucleophile scavengers, such as in the presence of reducing agents with a free thiol group (10 mM dithiothreitol)^21^.

Intriguingly, the FrpC_1-414_ fragment exhibits a very high affinity (K_D_ ~ 0.2 nM) for FrpD, the other protein expressed from the iron-regulated *frpDC* operon^22^. FrpD is a highly conserved lipoprotein located in the outer membrane of all meningococcal strains, and it does not exhibit similarity to known protein sequences from other organisms. FrpD is translated as a 271-residue polypeptide, of which the first 24 residues constitute a type II signal peptide that is processed after protein export across the cytoplasmic membrane. In the periplasm, the FrpD polypeptide is covalently modified on the Cys_25_ residue by a lipid moiety, and the mature FrpD lipoprotein is sorted to the outer membrane of the bacterium^23^. The biological function of the FrpD lipoprotein is unknown, but it was speculated that it might serve as an accessory lipoprotein tethering the liberated FrpC_1-414_ fragment to the bacterial cell surface.

In the present work, we defined the structure-function relationships underlying the interaction between the FrpD and FrpC_1-414_ proteins. We determined a low-resolution structure of the FrpD-FrpC_1-414_ complex in solution by small angle X-ray scattering (SAXS). The binding interface of FrpD was identified by chemical cross-linking and NMR chemical shift assays. These data were employed to localize the FrpD subunit within the complex by superimposing a newly resolved FrpD crystal structure onto the SAXS model of the FrpD-FrpC_1-414_ complex.

## Results

### Determination of the FrpD crystal structure

The FrpD_43-271_ protein (FrpD), a construct lacking the first 42 residues encompassing the N-terminal signal peptide (residues 1–24) and the linker sequence (residues 24–43), has been previously characterized^24^. The protein crystallized in two alternative crystal forms, belonging to the hexagonal space group *P*6_4_ and the orthorhombic space group *P*2_1_2_1_2_1_, both accommodating a single molecule in the asymmetric unit. Experimental phases were obtained from single-wavelength anomalous dispersion (SAD) of crystals of selenomethionine-labelled FrpD protein (SeMet FrpD) grown in the orthorhombic space group. The structure of the SeMet FrpD protein was refined to 1.4 Å resolution with an R value of 16.8% (R_free_ of 19.3%). The structure of native FrpD was determined at 2.3 Å resolution by molecular replacement using the SeMet FrpD structure and refined to an R value of 18.3% (R_free_ of 24.9%) (Table 1 and Supplementary Table S1). The refined crystallographic models of FrpD comprise 224 of the 229 residues, with one N-terminal and four C-terminal residues being disordered. Both FrpD structures are largely identical, with a coordinate root-mean-square deviation (rmsd) of 0.56 Å over the 224 aligned residues.

### FrpD adopts a novel fold

**Figure 2:**
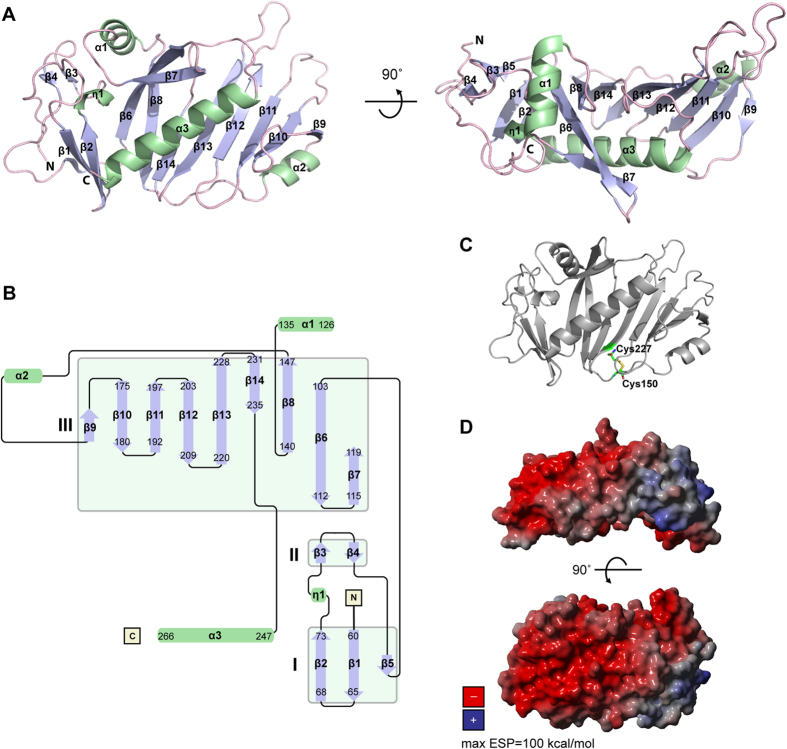
Structure of the *N. meningitidis* FrpD lipoprotein. (**A**) Ribbon representation of the FrpD structure: α helices and β strands are numbered consecutively and coloured in green and blue, respectively. (**B**) Topology of FrpD secondary structure elements. (**C**) Localization of the disulfide bond between the Cys_150_ and Cys_227_ residues in FrpD. (**D**) Electrostatic surface potential (ESP) of FrpD. Positive and negative potentials are shown in blue and red, respectively. The contouring values range from −100 to +100 kcal/mol.

FrpD displays a compact, slightly concave bean-shaped structure and consists of fourteen β strands (β1–β14), one 3_10_ helix (η1), two short α helices (α1, α2), and one long C-terminal helix α3 (Fig. 2A). The FrpD fold comprises three blocks of β sheets (I-III, Fig. 2B) flanked by helices. Block I is an antiparallel β sheet composed of the β1, β2 and β5 strands; block II contains two antiparallel β strands (β3 and β4); and block III is a nine-stranded antiparallel β sheet composed of the β6 to β14 strands. The order of secondary structural elements is β1–β2*–*η1–β3*–*β4–β5–β6–β7–α1–β8–α2–β9–β10–β11–β12–β13–β14–α3 (Fig. 2B). The structure comprises one β–3_10_–β motif (β2*–*η1*–*β3), two β–α–β motifs (β7–α1–β8 and β8–α2–β9), and eight β hairpins (β1–β2, β3–β4, β6–β7, β9–β10, β10–β11, β11–β12, β12–β13, β13–β14). A single disulphide bond in a right-handed spiral conformation is present between the Cys_150_ and Cys_227_ residues (Fig. 2C). The electrostatic surface potential map revealed that the FrpD surface is highly negatively charged, except for a single positively charged region located at the β9 and β10 strands of the structure (Fig. 2D).

**Figure 3:**
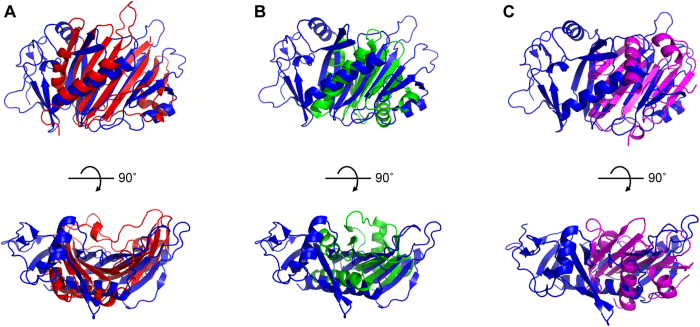
FrpD has no structural homologs within the PDB. Overlay of the FrpD structure (blue) with the uncharacterized Rv0999 ortholog protein (red, PDB entry code 4TMD) from *M. smegmatis* (**A**), chain B of yeast profilin (green, PDB entry code 3D9Y) from *Schizosaccharomyces pombe* (**B**), and chain B of the Mog1p-fold protein (magenta; PDB entry code 3V7B) from *Corynebacterium diphtheriae* (**C**).

Structural similarity searches revealed that none of the deposited structures in the Protein Data Bank (PDB) is substantially similar to the FrpD structure. Using the DALI server, the best fit was obtained for the crystal structure of the hypothetical protein MSMEI_5302 from *Mycobacterium smegmatis* (PDB: 4TMD), an Rv0999 orthologue of a *Mycobacterium tuberculosis* surface lipoprotein of unknown function (Fig. 3A). The sequence similarity was very weak (9%), and the structural alignment had a Z*-*score of 5.2 and rmsd of 3.8 Å for 114 aligned residues. A structural alignment search using PDBeFold showed structural similarity between FrpD and DIP2269 (PDB: 3V7B), a protein from *Corynebacterium diphtheria* of unknown function (Fig. 3B). Similarly, the proteins shared a very low sequence identity (4%), and the protein structures had a Z*-*score of 4.7 and rmsd of 2.98 Å for 80 aligned residues. A hierarchic classification of protein domain structures using CATH revealed that the C-terminal segment of the FrpD structure is similar to dynein light chain 2a (CATH superfamily 3.30.450.30) from yeast profilin, an actin-binding protein involved in the dynamic turnover and restructuring of the actin cytoskeleton (Fig. 3C; PDB entry code 3D9Y^25^). However, the CATH analysis yielded a SSAP score of 72.6 with an rmsd of 6.8 Å for 126 residues, which indicates that the structural similarity of FrpD to yeast profilin is weak, and the proteins only share a common structural motif made by a bundle of antiparallel β strands flanked by the C-terminal α helix. Moreover, structural alignment of the FrpD structure and that of each of the best-scoring candidates using Combinatorial Extension (CE) displayed low Z-scores (<4.2) and high rmsd values (>3.9)^26^,^27^. Hence, the similarity of FrpD fold topology to other proteins was not sufficient for establishment of structural homology, indicating that FrpD possesses a novel fold.

### Preparation of the FrpD/FrpC_1–414_ complex

**Figure 4:**
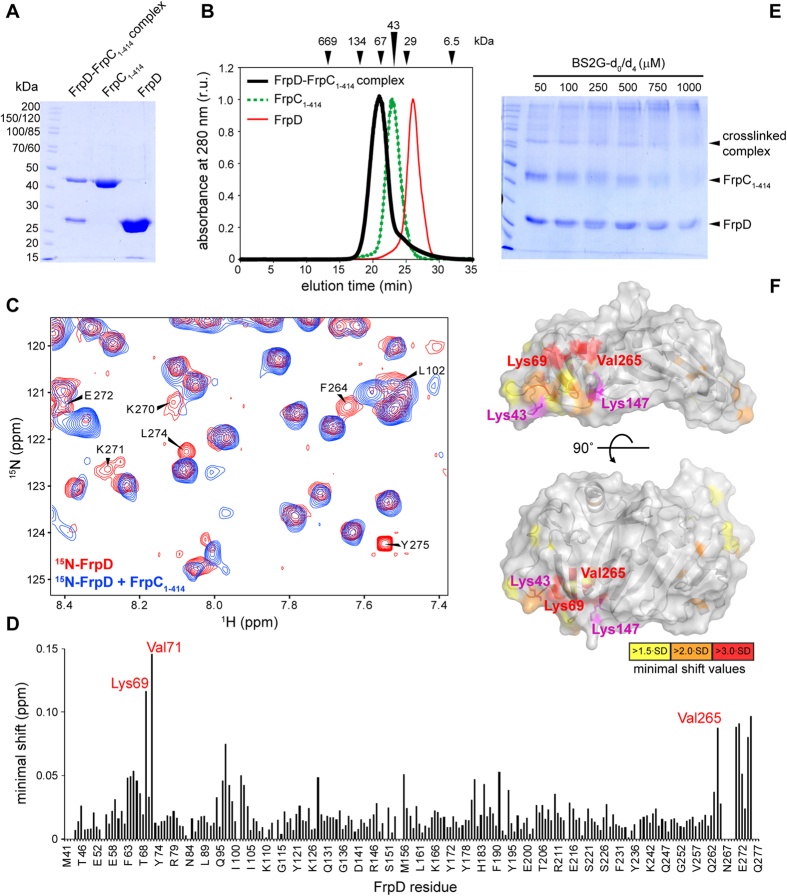
Identification of FrpD residues involved in FrpC_1-414_ binding. (**A**) SDS–PAGE analysis of purified FrpD-FrpC_1-414_ complex and free FrpD and FrpC_1-414_ proteins. Molecular weight markers are indicated on the left. (**B**) Size-exclusion chromatography of the FrpD-FrpC_1-414_ complex (black thick line), free FrpC_1-414_ (green dashed line) and free FrpD (red thin line). The calibration of the Superdex 200 HR column is indicated by arrowheads, with the following proteins used as standards: thyreoglobulin (669 kDa), bovine serum albumin (134 and 67 kDa), ovalbumin (43 kDa), carbonic anhydrase (29 kDa), and aprotinin (6.5 kDa). (**C**) NMR chemical shift perturbations of FrpD upon FrpC_1-414_ binding. The selected area of the overlay of the ^15^N-^1^H HSQC spectra of ^15^N-labelled FrpD in the absence (red) and presence (blue) of unlabelled FrpC_1-414_. The significantly perturbed residues are indicated. (**D**) Minimal backbone chemical shift values (^15^N and ^1^H^N^) of the assigned FrpD residues upon FrpC_1-414_ binding. (**E**) SDS–PAGE analysis of proteins obtained after chemical cross-linking of the FrpD-FrpC_1-414_ complex (1 mg/ml) with a 1:1 molar mixture of the nondeuterated (d_0_) and deuterated (d_4_) amine-reactive cross-linker bis(sulfosuccinimidyl) 2,2,4,4-glutarate (BS2G). (**F**) Localization of the perturbed residues upon FrpC_1-414_ binding on the surface of the free FrpD structure. The residues are coloured from yellow to red according to the extent of perturbation. The lysine residues (Lys43 and Lys 147) involved in the intermolecular crosslinks with FrpC_1-414_ are coloured in magenta.

Prochazkova *et al*.^22^ showed that FrpD binds the N-terminal segment of the FrpC protein with very high affinity (*K*_*d*_ ~ 0.2 nM). To characterize the FrpD-FrpC interaction at a structural level, we prepared a stable complex of the N-terminal domain of FrpC with FrpD. The N-terminal domain of FrpC, corresponding to residues 1–414 (FrpC_1-414_), was obtained as a 42-kDa fragment from the Ca^2+^-dependent cleavage of the purified FrpC_1-862_ protein^18^,^19^. The FrpC_1-414_ polypeptide was purified by a combination of nickel affinity and ion-exchange chromatography under denaturing conditions in 8 M urea and it was refolded by rapid dilution in ice-cold aqueous buffer (Fig. 4A). This procedure yielded monomeric FrpC_1-414_, as confirmed by a single peak on a size exclusion column, with the retention time corresponding to a monomeric form of the protein (~45 kDa, Fig. 4B). The FrpD/FrpC_1-414_ complex was prepared by mixing equimolar amounts of the purified FrpD and FrpC_1-414_ proteins. The FrpD/FrpC_1-414_ mixture eluted as a single peak with the retention time corresponding to an apparent molecular mass of about 70 kDa, indicating that the FrpD/FrpC_1-414_ complex is a monomeric protein complex with a 1:1 subunit stoichiometry (Fig. 4B).

### Mapping the FrpD/FrpC_1-414_ binding interface

The location of the FrpC_1-414_ binding site on FrpD was mapped by following the changes in the positions of backbone amide NMR signals of FrpD (^15^N and ^1^H) induced by the binding of FrpC_1-414_. Comparison of the ^1^H-^15^N heteronuclear single quantum coherence (HSQC) spectra of free ^15^N-FrpD and ^15^N-FrpD complexed with unlabelled FrpC_1-414_ revealed that the binding of FrpC_1-414_ results in significant line broadening, shifts in the positions and disappearance of certain FrpD cross-peaks (Supplementary Fig. S1). Based on our complete sequence-specific backbone resonance assignments for FrpD^28^, a minimal shift approach was used to assess the changes in the FrpD backbone amide signals after FrpC_1-414_ binding. In minimal shift approach, changes in chemical shift are usually ascribed by linking each resonance of free protein to the signal in the spectrum of a complex that has moved the least from the position of the free protein^29^. Significantly affected backbone amide signals of FrpD are highlighted in Fig. 4C. The minimal shift values were calculated for all the assigned FrpD residues and plotted as histogram against the protein sequence (Fig. 4D). FrpD residues with significantly perturbed backbone amides were localized, in part, at the surface-exposed portions of the β1, β2, and β5 strands at the N-terminus of the polypeptide, and at the C-terminal segment of the α3 helix and the unstructured tail at the C-terminus of FrpD. In particular, the following residues form a continuous binding surface: Phe63, Asp64, and Phe65 of the β1strand; Gln66 and Gly67 of the β1/β2 connecting loop; Lys69 and Val71 of the β2 strand; Asp96 and Ala97 of the β4/β5 connecting loop; Tyr98 of the β5 strand; Leu102 and Ile103 of the β5/β6 connecting loop; Phe264 of helix α3; and Lys270, Lys271, Glu272, Leu274, and Tyr275 at the C-terminus of FrpD (Fig. 4F).

To further analyse the FrpD/FrpC_1-414_ binding interface, we performed chemical cross-linking of primary amines in the FrpD/FrpC_1-414_ complex using a 1:1 molar mixture of the nondeuterated and deuterated (d_0_/d_4_) amine-reactive cross-linker bis-sulfosuccinimidyl glutarate (BS2G). The use of d_0_/d_4_ cross-linkers usually results in cross-linked peptides characterized by their distinct doublet isotope pattern with a mass difference of 4.025 Da in the deconvoluted mass spectra^30^. At low concentration of BS2G (50 μM), chemical cross-linking of the FrpD/FrpC_1-414_ complex (14 μM) resulted predominantly in the appearance of a single band with a molecular weight of approximately 70 kDa, corresponding to a monomeric complex with 1:1 subunit stoichiometry (Fig. 4E). In contrast, high concentrations of the cross-linker gave rise to high-molecular protein bands (>200 kDa), which most likely represent nonspecific aggregates of proteins. Thus, only the 70-kDa cross-link product was excised from the gel, digested with trypsin and the generated peptides were separated using a μHPLC system coupled online to an electrospray ionization Fourier transform ion cyclotron resonance (ESI FTICR) mass spectrometer. The high mass accuracy (better than 2 ppm) was sufficient to provide unambiguous assignments of cross-links using the MSlinks algorithm^31^. The cross-linked residues derived from the identified cross-linked peptides are listed in Supplementary Table S2. Out of nine cross-links identified, two and four were found as intramolecular crosslinks in FrpD and FrpC_1-414_, respectively (Supplementary Fig. S2). Intriguingly, the inter-residue distance constraints (BS2G spacer arm of 7.7 Å) of the cross-linked FrpD lysine residues (K166/K181 and K198/K167) corresponded well to the inter-residue distances (9.2 and 9.6 Å) of these lysine side chains in the crystal structure of FrpD, indicating a good specificity of the cross-linking reaction. Moreover, three intermolecular cross-links between the individual subunits were identified in the FrpD-FrpC_1-414_ complex (Supplementary Fig. S2). The FrpD lysine residue K43 was found to cross-link with two FrpC_1-414_ lysine residues (K74 and K267), while the FrpD lysine residue K147 was crosslinked to the K74 residue of FrpC_1-414_. In contrast to the K69 residue of FrpD that is directly involved in FrpC_1-414_ binding (Fig. 4D), the cross-linked K43 and K147 residues of FrpD are indeed located in close proximity to the FrpC_1-414_ binding site (Fig. 4F), but appear not to participate directly in the FrpC_1-414_ binding due to lack of chemical shift perturbation of the K147 signal (Fig. 4D). Taken together, the FrpC_1-414_ binding site on FrpD was unambiguously determined, indicating that chemical shift mapping and cross-linking experiments provide complementary results.

### Low-resolution structure of FrpD and the FrpD-FrpC_1-414_ complex

**Figure 5:**
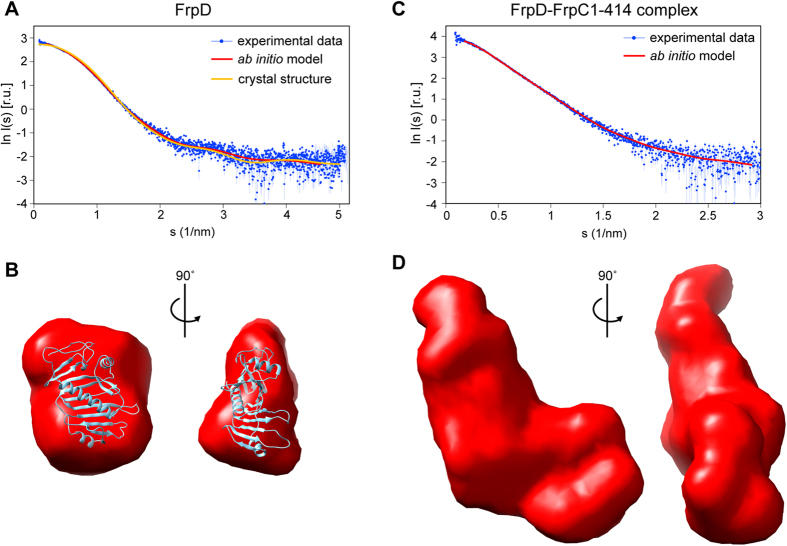
Low resolution structures of FrpD and the FrpD-FrpC_1-414_ complex. The SAXS plots of FrpD (**A**) and the FrpD-FrpC_1-414_ complex (**B**). The SAXS structural model of FrpD superimposed with the crystal structure of FrpD. (**C**) Experimental curves are shown as blue dots with error bars, and the theoretical scattering intensities derived from the ab initio models are given as red lines. The orange line represents the theoretical scattering curve generated for the FrpD crystal structure (PDB ID: 5EDJ). (**D**) The SAXS structural model of the FrpD-FrpC_1-414_ complex.

Low-resolution structures of FrpD and the FrpD-FrpC_1-414_ complex in solution were obtained by small angle X-ray scattering (SAXS). Overall parameters of the SAXS experiments, derived from experimental scattering curves, are presented in Supplementary Table S3. The experimental scattering curve for FrpD is shown in Fig. 5A. The values of the radius of gyration calculated from the Guinier plot (R_g_ = 22 Å), the maximum size of the particle derived from the pair-distance distribution function (D_max_ = 65 Å), and the molecular mass estimated from the Porod volume (25 kDa) were indicative of a well-folded and compact object. As expected, the values corresponded well to monomeric FrpD. Moreover, the SAXS-derived *ab initio* model of FrpD had a compact, slightly bent structure that fits the X-ray crystal structure of FrpD with a normalized spatial discrepancy of 0.9 and χ^2^ = 1.3 (Fig. 5B).

The structural parameters of the FrpD-FrpC_1-414_ complex were determined from the experimental scattering curve of the complex (Fig. 5C). The molecular mass of the FrpD-FrpC_1-414_ complex estimated from the Porod volume was 74 kDa, which corresponds well to the expected size of a monomeric complex with a 1:1 subunit stoichiometry (73 kDa). The R_g_ and D_max_ values of the FrpD-FrpC_1-414_ complex (37 Å and 130 Å) were larger than those of FrpD, indicating a compact complex structure. The excluded volume of the hydrated particle (Porod volume) was 123,000 Å^3^, and the calculated dry volume was 94,200 Å^3^, which is indicative of an elongated particle with uniform protein density. The low-resolution *ab initio* model of the FrpD-FrpC_1-414_ complex possesses an elongated shape, with one end broader than the other (Fig. 5D). The size of the broader part of the complex corresponds well with the size of FrpD, suggesting that the FrpD subunit may form the broader part of the complex.

### Biological function of the FrpD-FrpC_1-414_ complex

**Figure 6:**
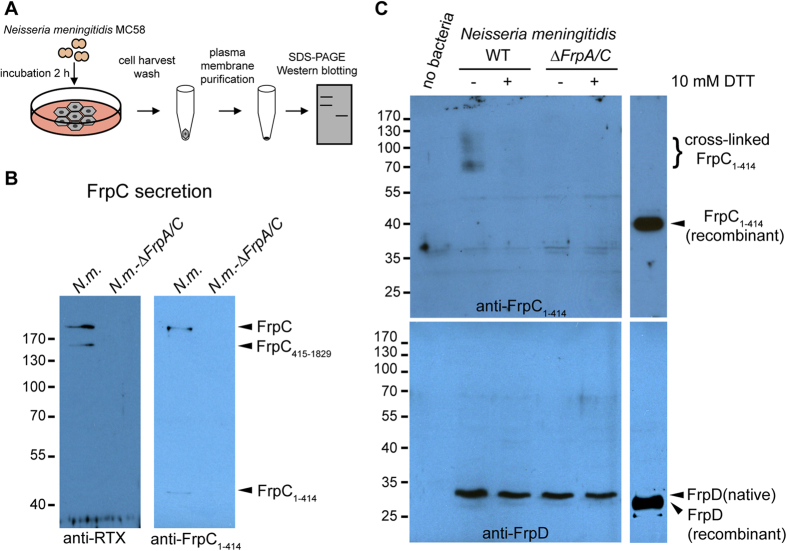
Ca^2+^-dependent *Trans*-splicing activity of FrpC results in covalent binding of FrpC_1-414_ to epithelial cells *in vitro*. (**A**) Schematic representation of the experiment. A549 cells were incubated with *N. meningitidis* cells at a multiplicity of infection of 200. After 2 h, the culture supernatant (**B**) and plasma membranes of the host cells (**C**), purified from the cell extract of hypotonically lysed cells, were subjected to SDS-PAGE and Western blotting. (**B**) Immunochemical detection of FrpC and FrpC_1-414_ in the culture supernatant of *N. meningitidis*-infected cells. A549 cells were incubated with the wild-type (*N.m.*) or *frpA/C*-deficient strain of *N. meningitidis* MC58 (*N.m.-ΔFrpA/C*), and proteins from the culture supernatant resolved by SDS-PAGE were probed either with the monoclonal 9D4 antibody recognizing the C-terminal RTX domain of FrpC (anti-RTX, left panel) or the rabbit polyclonal serum raised against the N-terminal domain of FrpC, FrpC_1-414_ (anti-FrpC_1-414_, right panel). (**C**) Immunochemical detection of FrpC_1-414_ (upper panel) and FrpD (middle panel) associated with the plasma membrane fraction of A549 cells after 2-h incubation with the wild-type or *frpA/C*-deficient strain of *N. meningitidis* in the presence or absence of 10 mM DTT. Purified plasma membranes were resolved by SDS-PAGE and probed with anti-FrpC_1-414_ serum (upper panel) or rabbit polyclonal serum raised against the purified FrpD_22-271_ protein (middle panel). Immunodetection of the recombinant FrpC_1-414_ and FrpD proteins is indicated on the right. The data are representative of three independent experiments.

Following processing of the freshly secreted FrpC in the Ca^2+^-rich body fluids by the *trans*-splicing reaction, the FrpC_1-414_ fragment is generated that bears a highly reactive anhydride at the C-terminal D_414_ residue, which might form covalent isopeptide bonds to surface proteins of epithelial cells. Given that FrpD is an outer membrane surface lipoprotein of *N. meningitidis* that exhibits a high affinity to the FrpC_1-414_ fragment, we tested *in vitro* the hypothesis that formation of the FrpD-FrpC_1-414_ complex might be involved in binding of *N. meningitidis* to the target cell surface. Human alveolar epithelial cells (A549) were exposed to the wild-type strain of *N. meningitidis* MC58, or to the *frpA/C* double knock-out mutant (an MC58-derived strain having the genes encoding the prototypical FrpC protein (NMB1415) and the paralogous FrpA protein (NMB0585) deleted). The crosslinking of the FrpC_1-414_ fragment to human epithelial cell surface upon co-incubation with the two strains was then assessed using purified plasma membranes of A549 airway epithelial cells (Fig. 6A).

First, we examined the secretion of FrpC into culture supernatants by Western blotting using the 9D4 monoclonal antibody, which recognizes the C-terminal RTX repeats of FrpC (anti-RTX), and FrpC_1-414_-specific rabbit serum (anti-FrpC_1-414_) raised against the purified FrpC_1-414_ protein. As shown in Fig. 6B, the anti-RTX antibody detected in the wild-type strain two bands of approximately 200 and 150 kDa, which corresponds well to the size of full-length FrpC and its processed 1415-residue C-terminal segment (FrpC_415-1829_). Additionally, the anti-FrpC_1-414_ serum recognized both full-length FrpC (200 kDa) and free FrpC_1-414_ (45 kDa), indicating the cleavage activity of freshly secreted FrpC in the culture supernatants. In contrast, no bands were detected in the culture supernatant of the *frpA/C*-deficient strain, demonstrating the high specificity of the anti-FrpC_1-414_ serum.

To analyse the capacity of the liberated FrpC_1-414_ to covalently link to surface proteins of epithelial cells, we exposed monolayers of the A549 cells to the wild-type and *frpA/C*-deficient strains in the presence or absence of 10 mM dithiothreitol (DTT), a reducing agent that completely inhibits the cross-linking activity of FrpC^21^. After two hours of incubation, the purified plasma membranes of the A549 cells were resolved on SDS-PAGE and probed on immunoblots with the anti-FrpC_1-414_ serum. As shown in Fig. 6C, the anti-FrpC_1-414_ serum detected several bands (approximately 30, 37, and 50 kDa) that were present in both treated and untreated cells, indicating some cross-reactivity of the serum antibodies with plasma membrane proteins of the A549 cells. However, the immunoblot also revealed several bands ranging from 70 kDa to 130 kDa that were exclusively detected in the plasma membrane fraction of A549 cells exposed to the wild-type *N. meningitidis* in the absence of 10 mM DTT. No such bands were observed in the presence of 10 mM DTT for the wild-type or in the absence or presence of 10 mM DTT for the *frpA/C*-deficient strain, indicating that the 70–130 kDa bands likely represent FrpC_1-414_ cross-linked to specific membrane proteins. These results clearly suggest that the *trans*-splicing activity of FrpC occurs not only at the level of purified proteins but also during bacterial adhesion onto epithelial cells *in vitro*. Moreover, the amount of bacteria adhered to the plasma membranes was evaluated by detection with rabbit polyclonal serum raised against purified FrpD_22-271_^22^. As shown in Fig. 6C, the anti-FrpD serum recognized a single band with a molecular size of approximately 30 kDa, which is slightly higher than that of the crystallized FrpD_43-271_ protein, but corresponds well to native FrpD (FrpD_25-271_) carrying a lipid moiety. Quantification of the immunoblots revealed that the FrpD intensity associated with plasma membranes of A549 cells exposed to the wild-type strain was slightly decreased in the presence of 10 mM DTT and remained at similar levels even for cells treated with the *frpA/C*-deficient mutant, regardless of the presence or absence of 10 mM DTT (Fig. 6C). However, differences between the FrpD intensities were not statistically significant. Taken together, these results demonstrate that formation of the FrpD-FrpC_1-414_ complex may be involved in the interaction of *N. meningitidis* with epithelial cells.

## Discussion

Adhesion of *N. meningitidis* to the mucosal epithelia of the human nasopharynx represents the first step of host colonization and subsequent development of meningococcal pathogenesis. Adhesion is a dynamic process resulting from a balance of interactions between the bacterial and host cell surface structures. Type IV pili are regarded as the major *Neisseria* adhesins, and rapid elongation of these polymeric filaments on the bacterial surface allows the initial attachment to the mucosal epithelia. Once the primary bacteria-cell contact is established, the pili are retracted, and a different repertoire of other adhesins orchestrates a deeper interaction with the host cell surface. In addition to the outer membrane opacity proteins (Opa and Opc), which promote the intimate adhesion and invasion of host cells, several minor adhesins, including *Neisseria* hia/hsf homologue A (NhhA), adhesion and penetration protein (App), *Neisseria* adhesin A (NadA), and meningococcal serine protease A (MspA), mediate binding to epithelial cells. Here, we demonstrate the structural basis of the interaction between the outer membrane FrpD lipoprotein and FrpC_1-414_, which covalently links to epithelial cells after the Ca^2+^-induced *trans*-splicing of freshly secreted FrpC. Although the atomic structure of FrpC_1-414_ remains unknown, formation of the FrpD-FrpC_1-414_ complex may potentially represent a conceptually novel adhesion strategy used by *N. meningitidis* during colonization of the respiratory epithelia.

**Figure 7:**
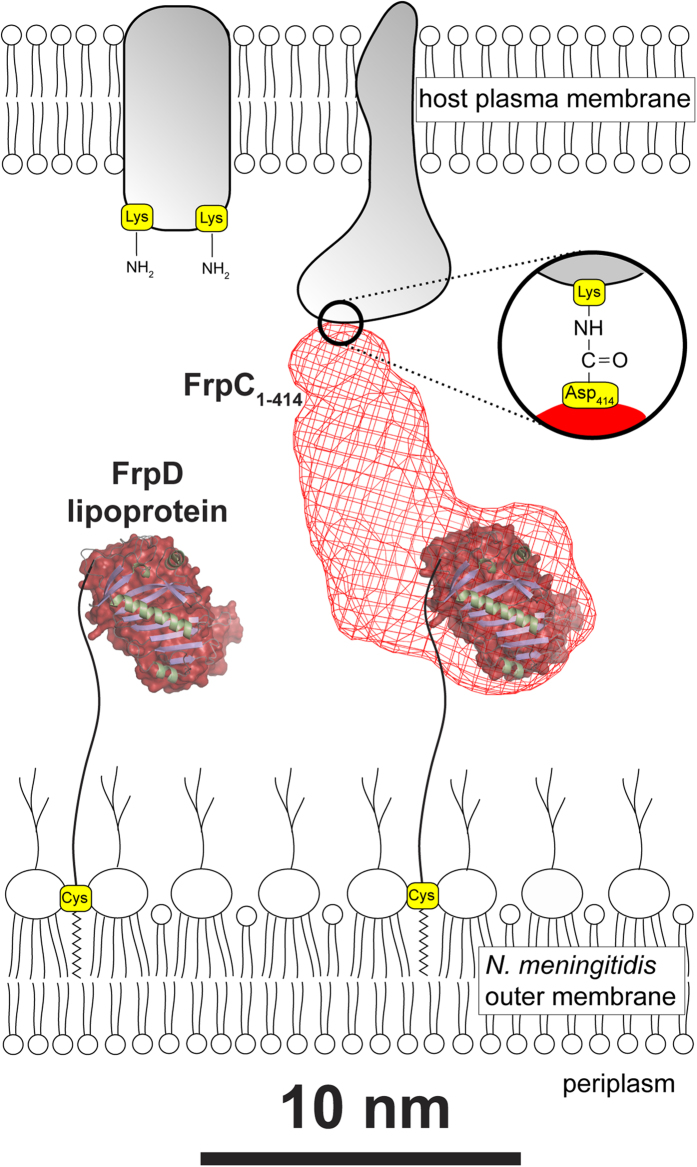
Formation of the FrpD-FrpC_1-414_ complex may be involved in the interaction of *N. meningitidis* with the target host cell surface. FrpD is a highly conserved lipoprotein that is anchored to the outer membrane of *N. meningitidis* through covalent lipidation of the Cys_25_ residue. The N-terminal portion of FrpD (residues 25–42) forms a flexible linker that traverses the oligosaccharide layer of the outer membrane lipooligosaccharide and links the globular domain of FrpD (residues 43–271) to the bacterial cell surface. FrpD binds with high affinity the N-terminal domain of FrpC (FrpC_1-414_), which can be covalently attached to surface proteins of the host cells *via* an Asp-Lys isopeptide bond after being released from secreted FrpC by the Ca^2+^-dependent autocatalytic processing. Thus, formation of the FrpD-FrpC_1-414_ complex may be involved in the interaction of *N. meningitidis* with mucosal surfaces.

FrpD is a highly conserved lipoprotein that is anchored to the outer membrane of meningococci *via* covalent lipidation of the Cys_25_ residue, which becomes exposed after processing of the signal peptide (the 24 N-terminal residues) of the FrpD precursor during protein sorting^22^. We have previously reported the purification and crystallization of the native and Se-Met derivative of the FrpD_43-271_ protein^24^. Here, we determined the crystal structure of the FrpD_43-271_ at 1.4 Å resolution. The structure is characterized by a C-terminal α-helix (α3) that traverses a series of antiparallel β-strands (Fig. 2A). Remarkably, this fold does not have any significant homology to known protein structures and it appears to be a unique fold that can be found in peripheral outer membrane lipoproteins of Gram-negative bacteria. The structure of the N-terminal part of FrpD, encompassing residues 25–42, remains unknown due to specific cleavage of the residues during protein crystallization^24^. In agreement with the secondary structure prediction showing that these residues do not have a propensity to adopt any secondary structure, the 25–42 peptide appears to form a flexible linker that connects the globular domain of FrpD to the lipid anchor through the oligosaccharide layer of the outer membrane lipooligosaccharide (Fig. 7).

Prochazkova *et al*.^22^ reported that FrpD has a very high affinity (K_D_ ~ 0.2 nM) to the N-terminal domain of FrpC. Both FrpD and FrpC are encoded with the same *frpDC* operon, which is transcribed under iron-limiting conditions. Such conditions prevail on the mucosal surfaces of the human nasopharynx and activate production of the FrpD and FrpC proteins during meningococcal colonization of host epithelia. Unlike FrpD, which is sorted to the outer membrane of the bacterium, FrpC is one of the few meningococcal proteins that are secreted into the extracellular space. FrpC is highly immunogenic and elicits high levels of both IgG and IgA class antibodies in convalescent-phase sera of patients 2 to 5 weeks after the first symptoms of meningococcal disease appear^13^. However, FrpC was shown to be dispensable for virulence in an infant rat model of infection, and its biological function remains unknown^16^,^17^.

Osicka *et al*.^18^ demonstrated that in the presence of Ca^2+^ ions the purified recombinant FrpC undergoes a highly specific processing that resembles protein *trans*-splicing. This involves Ca^2+^-dependent folding of the self-processing module (SPM) of FrpC that is associated with autocatalytic cleavage of the Asp_414_-Pro_415_ peptide bond and subsequent covalent linkage of the liberated carboxyl group of Asp_414_ of FrpC_1-414_ to an ε-amino group from a neighbouring lysine residue of another molecule^19^. Indeed, at the very low calcium concentrations within bacterial cytoplasm (<100 nM) FrpC remains in an unfolded conformation, which is essential for efficient translocation of the polypeptide through the T1SS conduit out of the bacterial cell^32^. Upon secretion of FrpC, the high concentrations of calcium ions in the host body fluid environment (>1 mM) induce the SPM-mediated cleavage and release of FrpC_1-414_ into mucosal fluids (42 kDa, Fig. 6B) and the subsequent covalent linkage of the released FrpC_1-414_ onto plasma membrane proteins of epithelial cells (70-130 kDa, Fig. 6C). Thus, the FrpC_1-414_ protein linked to the host cell surface potentially serves as a specific target for the binding of the FrpD lipoprotein that protrudes from the outer membrane of adhering bacterial cells (Fig. 7).

Unlike various types of weak interactions used by many pathogens to adhere to host cells, protein cross-linking does not appear to be a common adhesion strategy. However, *Candida albicans*, an opportunistic fungal pathogen that can cause severe oropharyngeal and oesophageal mucositis, hijacks host cell transglutaminases, which catalyse the formation of covalent N^ɛ^-(γ-glutamyl)lysine isodipeptide bonds between the surface-exposed Hwp1 (Hyphal wall protein 1) of fungal germ tubes and squamous epithelial cells of the oral cavity^33^. These bonds exhibit high resistance to proteolysis and enable penetration of emerging *C. albicans* hyphae within host tissues. In contrast, the *trans*-splicing activity of FrpC does not seem to serve as a major adhesion strategy for meningococcal cells, as FrpC appears to have a very small effect on overall adhesion to the epithelial cells *in vitro*. On the other hand, meningococci employ a broad range of adhesive molecules that orchestrate a close contact interaction of bacterial membrane with the host cell plasma membrane (i.e Opa proteins). Thus, the *trans*-splicing of FrpC could play a particular role not only in the attachment of bacteria to the host cell surface but also in traversal of the pseudostratified columnar epithelium of the upper respiratory tract^34^.

The primary amino acid sequence of SPM in FrpC is highly homologous to that of internal segments of very long RTX proteins (<1,000 residues)^19^. This indicates that Ca^2+^-dependent protein *trans*-splicing is not restricted only to FrpC of *N. meningitidis*, but appears to be widespread among other RTX proteins of Gram-negative pathogens that infect humans, animals, and plants. This is well-documented for the recombinant ApxIVA protein, a FrpC homolog of the animal pathogen *Actinobacillus pleuropneumoniae*, which undergoes Ca^2+^-dependent autocatalytic cleavage of the Asp_638_-Pro_639_ peptide bond and formation of higher molecular weight ApxIVA oligomers^18^. In contrast to ~110-kDa RTX pore-forming toxins of *A. pleuropneumoniae* (ApxI, ApxII, and ApxIII), which show strong hemolytic and cytotoxic activities against various cell types, the ~190-kDa ApxIVA protein does not seem to be cytotoxic, and its biological function remains unknown^35^. However, deletion of the *apxIVA* gene in *A. pleuropneumoniae* QP05 strain led to attenuation of *A. pleuropneumoniae* virulence in the experimental infection of piglets^36^. This could imply that the Ca^2+^-dependent *trans*-splicing activity of RTX proteins plays an important role in virulence of some Gram-negative pathogens.

## Methods

### Protein purification and crystallization

Native and SeMet FrpD proteins were expressed, purified, and crystallized as previously described^24^. FrpC_1-414_ was obtained as the 42-kDa fragment of the Ca^2+^-dependent cleavage of the purified FrpC_1-862_ construct^18^,^19^. The FrpC_1-862_ protein was expressed in *E. coli* BL21(λDE3) grown at 30 °C in LB medium supplemented with ampicillin (150 μg/ml). FrpC expression was induced with isopropyl-β-d-thiogalactopyranoside (1 mM). The cells were washed twice in TN buffer (50 mM Tris-HCl, pH 7.4, 150 mM NaCl) supplemented with 5 mM EDTA, resuspended in TN buffer, and disrupted by sonication. The cell lysate was cleared by centrifugation at 20,000 *g* for 30 min, and the supernatant was loaded onto a Ni-Sepharose column (GE Healthcare) equilibrated with TN buffer. The column was extensively washed with TN buffer supplemented with 30 mM imidazole, and the FrpC_1-862_ protein was eluted with TN buffer containing 200 mM imidazole. The eluted fractions were collected and supplemented with dithiothreitol (DTT) to a final concentration of 10 mM. The mixture then was dialyzed overnight at 4 °C in TN buffer containing 10 mM DTT and 10 mM CaCl_2_. Addition of Ca^2+^ ions induced the autocatalytic cleavage of FrpC_1-862_ into two protein products (FrpC_1-414_ and FrpC_415-862_). The presence of DTT prevented the cross-linking activity of FrpC_1-414_. The protein mixture was supplemented with urea to a final concentration of 8 M and loaded onto a Q-Sepharose column (GE Healthcare) equilibrated with 50 mM Tris-HCl (pH 7.4) and 8 M urea. FrpC_1-414_ was recovered from the column in the flow-through fraction and concentrated by ultrafiltration (Amicon, 10 K membrane). The denatured FrpC_1-414_ was refolded by rapid dilution in ice cold aqueous buffer containing 50 mM Tris-HCl (pH 7.4), 150 mM NaCl, and 2 mM CaCl_2_. The protein concentrations were measured by Bradford assay (Bio-rad).

### Structure Determination

Diffraction data were collected without any additional cryoprotection at 100 K at beamlines MX 14.1 and 14.2 of BESSY in Berlin, Germany^37^. Diffraction data were processed and scaled using the HKL-3000 software package^38^. The diffraction data for the native FrpD crystals were collected to a resolution of 2.3 Å using a wavelength of 0.918 Å. For the SeMet FrpD protein, two datasets were collected. The dataset used for SAD phasing (denominated SeMet1 in Table 1) was collected at 0.979 Å, and the crystal diffracted X-rays to a d_min_ of 2.0 Å. The dataset yielding 1.4 Å resolution (denominated SeMet2 in Table 1) was collected at a wavelength of 0.9184 Å. The diffraction properties of the crystals were significantly improved by a crystal annealing procedure, which was performed by blocking the cryostream for 3 s.

The crystal structure of the SeMet FrpD protein was determined by SAD utilizing the anomalous signal from selenium atoms. All procedures for phasing and phase improvement by density modification were carried out using the HKL-3000 software package^38^. The initial model, containing 95% of all residues, was constructed automatically by ARP/wARP^39^ using the electron density maps after density modification with the program DM^40^. The structure was refined to 1.4 Å using the high-resolution dataset of SeMet FrpD.

The crystal structure of native FrpD was solved by molecular replacement using MOLREP^41^. The crystals of native FrpD protein belonged to the hexagonal space group *P*6_2_ or its enantiomorph *P*6_4_. After the initial phase determination for both enantiomorphs, the *P*6_4_ enantiomorph was identified as the correct one. Model refinement was carried out using REFMAC 5.5^42^ and interspersed with manual adjustments using Coot^43^. The quality of the protein models was confirmed with MolProbity^44^ Electrostatic charge distribution was calculated by particle mesh Ewald approach implemented in YASARA^45^ with the Amber 96 force field. For calculation of the electrostatic surface, FrpD was protonated according to pH 7.4. The maximum ESP for the colour range was 100 kcal/mol. Comparative structure-based homology analysis of the FrpD structure was carried out using DALI^46^, CATH^47^, and PDBeFold^48^. Figures were prepared with PYMOL (http://www.pymol.org).

### Small-Angle X-ray Scattering (SAXS)

SAXS data were collected on the beamline X33 EMBL at DORIS-III in Hamburg, Germany^49^. The concentrations of FrpD and the FrpD-FrpC_1-414_ complex during the measurement were 1–9 mg/ml and 1–5 mg/ml, respectively. The molecular mass of the protein was estimated from forward scatter using a reference solution of bovine serum albumin (66 kDa). The data were recorded at room temperature using the pixel 1 M PILATUS detector (DECTRIS) at a sample-detector distance of 2.7 m, and a wavelength (λ) of 0.15 nm, covering the range of momentum transfer 0.06 nm^−1^ < s < 6 nm^−1^ (s = 4π sin(θ)/λ, where 2θ is the scattering angle). No radiation damage was observed during the data collection. The low-resolution structures of the proteins were reconstructed *ab initio* using DAMMIF^50^.

### Nuclear magnetic resonance (NMR)

^15^N-labelled FrpD was expressed and purified as previously described^28^. For preparation of the FrpD-FrpC_1-414_ complex, ^15^N-labelled FrpD was mixed with unlabelled FrpC_1-414_ in a 1:1 molar ratio, and the mixture was loaded onto a Superdex 200 HR gel filtration column equilibrated with 5 mM sodium phosphate, pH 7.2, containing 50 mM NaCl. The complex was concentrated by ultrafiltration, and the sample consisting of 450 μl of 0.5 mM protein, 10% D_2_O, and 0.05% NaN_3_ was used for the measurement. The NMR data were processed using Topspin 2.1 (Bruker) and analysed using SPARKY.

### Mapping of FrpC1-414 binding sites on FrpD

The minimal shift approach was used to assess the changes in the positions of FrpD signals resulting from FrpC_1-414_ binding. The minimum change in the position for all backbone amide peaks between the free and FrpC_1-414_-bound FrpD was obtained by using Microsoft Excel to calculate the combined chemical shift difference in ^15^N and ^1^H for each assigned peak in the ^15^N/^1^H HSQC spectrum of the free protein compared to all peaks observed in the HSQC spectra of the complexes formed with FrpC_1-414_. The combined amide proton and nitrogen chemical shift difference (Δδ) was defined according to the following equation:


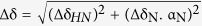


where Δδ_*HN*_ and Δδ_*N*_ correspond to the differences in ^1^H and ^15^N chemical shifts between pairs of compared HSQC peaks and α_*N*_ is a scaling factor of 0.2 required to account for differences in the range of amide proton and nitrogen chemical shifts. For each individual HSQC peak, the minimal shift induced by ligand binding was taken as the lowest possible combined shift value (Δδ). The resulting minimal shift values were plotted against the protein sequence. FrpD residues with significantly perturbed backbone signals were mapped onto the FrpD structure using PYMOL.

### Mass spectrometry

Aliquots of the FrpD-FrpC_1-414_ complex (1 mg/ml) were mixed with different concentrations of a 1:1 molar mixture of the nondeuterated (d_0_) and deuterated (d_4_) amine-reactive cross-linker bis(sulfosuccinimidyl) 2,2,4,4-glutarate (BS2G, Thermo Fisher Scientific). The cross-linking reactions were quenched after 30 min of incubation by adding ethanolamine to a final concentration of 1 mM. The proteins were resolved by SDS-PAGE and stained with Coomassie blue. The crosslinked 70-kDa band corresponding to the monomeric FrpD-FrpC_1-414_ complex was subjected to a nano-μHPLC/nano-ESI-FTICR mass spectrometry as previously described^51^. Briefly, the band was excised from the gel, destained and trypsinized overnight at 37 °C. The resulting peptides were desalted and loaded onto a C18 reversed-phase analytical column (Acclaim PepMap 100, Thermo Fisher Scientific) connected to an UltiMate 3000 RSLCnano System (Dionex, USA) and coupled to a SolariX XR FT-ICR mass spectrometer (Bruker Daltonics, Germany) equipped with a 12 T superconducting magnet. Mass spectral data were acquired in positive broadband mode over an *m*/*z* range of 245–2000, with 1 M data points transient, 0.4 s ion accumulation, and 4 scans accumulated per spectrum. Cross-links were identified using our self-written software based on the Links algorithm^31^. The algorithm identifies cross-linked peptides by matching experimental data to a theoretical library generated based on the protein sequence, protease specificity, cross-linker reactivity and composition, and protein chemical modification^51^.

### Cell cultures, bacterial strains, and growth conditions

A549 cells (ATCC CCL-185) were grown in DMEM supplemented with 2 mM glutamine and 10% fetal calf serum (Life Technologies) in humidified 5% CO_2_ at 37 °C. The wild-type and *frpA/C*-deficient strains of *Neisseria meningitidis* MC58 have been previously described^16^. The bacteria were grown on GC medium base agar (Difco) at 37 °C in 5% CO_2_. They were resuspended in DMEM (without fetal calf serum) at OD_600_ = 0.2 and cultivated under iron-depleted conditions with 100 μM desferrioxamine B at 37 °C^11^.

### Adherence assay

A549 cells were seeded into 6-well plates (10^5^ cells/well) and incubated with meningococci at a multiplicity of infection (MOI) of 200 in humidified 5% CO_2_ at 37 °C. After 2 h incubation, the aspirated culture supernatant was centrifuged at 18,000 g for 15 min, and the supernatant was mixed with SDS-PAGE loading buffer. The adherent cells were extensively washed three times with PBS and 0.1 M sodium acetate, pH 3.5, containing 150 mM NaCl. Cells were scraped and lysed with ice-cold hypotonic lysis buffer (10 mM Tris-HCl, pH 7.4) followed by multiple freeze/thaw cycles. The cell extracts were centrifuged at 18,000 g for 15 min and the pelleted material (crude membrane fraction) was resuspended in SDS-PAGE loading buffer. The proteins were separated on SDS-PAGE, transferred onto a nitrocellulose membrane, and probed with specific antibodies using Western blotting procedures.

## Additional Information

**Accession Codes:** Structure coordinates of the native and selenomethionine-derivative FrpD proteins are deposited in the PDB (http://www.rcsb.org) under accession codes 5EDJ and 5EDF, respectively. The SAXS models of FrpD and the FrpD-FrpC1-414 complex are deposited in the SASBDB (http://www.sasbdb.org) under codes SASDBP4 and SASDBQ4, respectively. The assigned backbone resonances for FrpD43-271 are deposited in the BMRB (http://www.bmrb.wisc.edu) under accession code 18779.

**How to cite this article:** Sviridova, E. *et al*. Structural basis of the interaction between the putative adhesion-involved and iron-regulated FrpD and FrpC proteins of *Neisseria meningitidis. Sci. Rep.* **7**, 40408; doi: 10.1038/srep40408 (2017).

**Publisher's note:** Springer Nature remains neutral with regard to jurisdictional claims in published maps and institutional affiliations.

## Supplementary Material

Supplementary Information

## Figures and Tables

**Figure 1:**
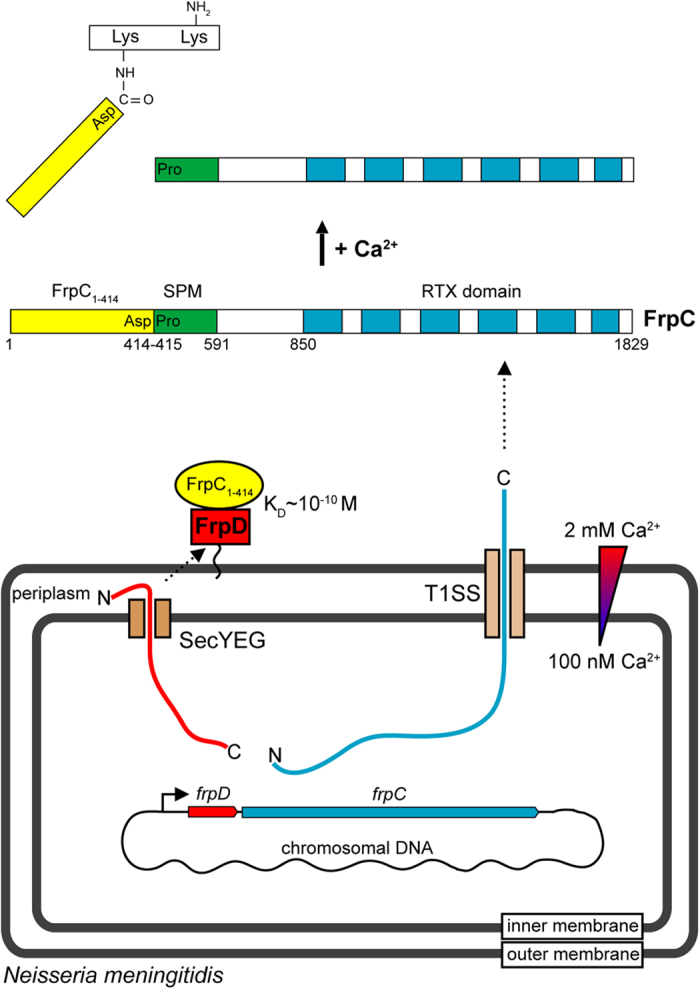
Localization of FrpD and FrpC proteins in *N. meningitidis* and Ca^2+^-dependent *trans*-splicing of secreted FrpC. The FrpD and FrpC proteins of the Gram-negative bacterium *N. meningitidis* are consecutively encoded in the *frpDC* operon, which is under control of the iron-regulated Fur promoter sequence. FrpD is translated in the bacterial cytoplasm as a pre-prolipoprotein containing an N-terminal signal peptide that is processed into a prolipoprotein after polypeptide export by the general secretory pathway (SecYEG). In the periplasmic space, the prolipoprotein matures into lipoprotein by covalent attachment of a lipid moiety, and the mature lipoprotein is sorted into the outer membrane of the bacterium (red rectangle). FrpC is translated as an unfolded polypeptide with a noncleavable C-terminal secretion signal that enables direct translocation (secretion) of the polypeptide through the Type 1 secretion system (T1SS). Binding of extracellular Ca^2+^ ions promotes folding of a self-processing module (SPM, in green) of FrpC that is associated with autocatalytic cleavage of the Asp_414_-Pro_415_ peptide bond and subsequent covalent linkage of the FrpC_1-414_ product (in yellow) to another protein molecule *via* a newly-formed Asp-Lys isopeptide bond.

**Table 1:** Crystallographic statistics from the SeMet derivative and native FrpD data collection.

Data set	SeMet1 phasing			Native		SeMet2 refinement		
Wavelength (Å)	0.9790			0.9180		0.9184		
Resolution range (Ǻ)^a^	50–2.00 (2.07–2.00)			50–2.30 (2.38–2.30)		50–1.40 (1.45–1.40)		
**Unit cell**								
a, b, c (Å)	38.1, 38.7, 165.5			115.4, 115.4, 38.8		38.3, 38.8, 165.7		
α, β, γ°	90, 90, 90			90, 90, 120		90, 90, 90		
Space group	*P*2_1_2_1_2_1_			*P*6_4_		*P*2_1_2_1_2_1_		
Multiplicity	5.9 (4.3)			6.9 (4.9)		5.7 (4.5)		
Unique reflections	100435			13324 (1247)		47065 (3963)		
Completeness (%)	98.2 (88.1)			99.3 (95.1)		94.2 (80.9)		
R_merge_^b^ (%)	5.5 (11.9)			7.1 (50.4)		4.7 (31.9)		
Average I/σ(I)	77.4 (20.0)			22.9 (2.45)		37.6 (2.7)		
**Phasing**								
Dataset used for phasing	SeMet1							
No. of Se sites (expected/found)	4/4							
Resolution shell limit (Å)	12.91	7.07	5.00	3.83	3.13	2.64	2.27	2.00
Phasing power^c^	2.68	2.79	2.63	1.72	1.76	1.72	1.29	0.76
R_cullis_^d^	0.34	0.32	0.34	0.48	0.47	0.48	0.58	0.79

^a^Statistics for the highest resolution shell are in parentheses.

^b^R_merge_ = (|I_hkl_ − 〈I〉|)/I_hkl_, where the average intensity 〈I〉 is taken over all symmetry equivalent measurements, and I_hkl_ is the measured intensity for any given reflection.

^c^Phasing power = ∑|F_H_|_calc_/∑||F_PH_|_obs_ − |F_PH_|_calc_|, where |F_H_|_calc_ is the calculated structure factor amplitude for the heavy atom model, and |F_PH_|_obs_ and |F_PH_|_calc_ are the observed and calculated structure factor amplitudes for the derivative.

^d^R_cullis_ = ∑||F_PH_ + F_P_| − |F_H_|_calc_|/∑|F_PH_ − F_P_|, where |F_H_|_calc_ is the calculated structure factor amplitude for the heavy atom model, and F_PH_ and F_P_ are the observed structure factors for the derivative and native protein, respectively.

## References

[b1] Pizza M. & Rappuoli R. *Neisseria meningitidis*: pathogenesis and immunity. Curr. Opin. Microbiol. 23, 68–72 (2015). 25461575 10.1016/j.mib.2014.11.006

[b2] Rosenstein N. E., Perkins B. A., Stephens D. S., Popovic T. & Hughes J. M. Meningococcal disease. N. Engl. J. Med. 344, 1378–1388 (2001). 11333996 10.1056/NEJM200105033441807

[b3] Rouphael N. G. & Stephens D. S. *Neisseria meningitidis*: biology, microbiology, and epidemiology. Methods Mol. Biol. 799, 1–20 (2012). 21993636 10.1007/978-1-61779-346-2_1 PMC4349422

[b4] Stephens D. S., Greenwood B. & Brandtzaeg P. Epidemic meningitis, meningococcaemia, and *Neisseria meningitidis*. Lancet 369, 2196–2210 (2007). 17604802 10.1016/S0140-6736(07)61016-2

[b5] Hill D. J., Griffiths N. J., Borodina E. & Virji M. Cellular and molecular biology of *Neisseria meningitidis* colonization and invasive disease. Clin. Sci. 118, 547–564 (2010). 20132098 10.1042/CS20090513 PMC2830671

[b6] Capecchi B. . *Neisseria meningitidis* NadA is a new invasin which promotes bacterial adhesion to and penetration into human epithelial cells. Mol. Microbiol. 55, 687–698 (2005). 15660996 10.1111/j.1365-2958.2004.04423.x

[b7] Mattick J. S. Type IV pili and twitching motility. Annu. Rev. Microbiol. 56, 289–314 (2002). 12142488 10.1146/annurev.micro.56.012302.160938

[b8] Sadarangani M., Pollard A. J. & Gray-Owen S. D. Opa proteins and CEACAMs: pathways of immune engagement for pathogenic *Neisseria*. FEMS Microbiol Rev. 35, 498–514 (2011). 21204865 10.1111/j.1574-6976.2010.00260.x

[b9] Scarselli M. . *Neisseria meningitidis* NhhA is a multifunctional trimeric autotransporter adhesin. Mol. Microbiol. 61, 631–644 (2006). 16803596 10.1111/j.1365-2958.2006.05261.x

[b10] Grifantini R. . Identification of iron-activated and –repressed Fur-dependent genes by transcriptome analysis of *Neisseria meningitidis* group B. Proc. Natl. Acad. Sci. USA 100, 9542–9547 (2003). 12883001 10.1073/pnas.1033001100 PMC170954

[b11] Basler M. . The iron-regulated transcriptome and proteome of *Neisseria meningitidis* serogroup C. Proteomics 6, 6194–6206 (2006). 17133369 10.1002/pmic.200600312

[b12] Thompson S. A., Wang L. L., West A. & Sparling P. F. *Neisseria meningitidis* produces iron-regulated proteins related to the RTX family of exoproteins. J. Bacteriol. 175, 811–818 (1993). 8423153 10.1128/jb.175.3.811-818.1993 PMC196221

[b13] Osicka R., Kalmusova J., Krizova P. & Sebo P. *Neisseria meningitidis* RTX protein FrpC induces high levels of serum antibodies during invasive disease: polymorphism of frpC alleles and purification of recombinant FrpC. Infect. Immun. 69, 5509–5519 (2001). 11500424 10.1128/IAI.69.9.5509-5519.2001 PMC98664

[b14] Linhartova I. . RTX proteins: a highly diverse family secreted by a common mechanism. FEMS Microbiol. Rev. 34, 1076–1112 (2010). 20528947 10.1111/j.1574-6976.2010.00231.x PMC3034196

[b15] Bumba L. . Calcium-driven folding of RTX domain β-rolls ratchets translocation of RTX proteins through Type I secretion ducts. Mol. Cell 62, 47–62 (2016). 27058787 10.1016/j.molcel.2016.03.018

[b16] Forman S. . *Neisseria meningitidis* RTX proteins are not required for virulence in infant rats. Infect. Immun. 71, 2253–2257 (2003). 12654851 10.1128/IAI.71.4.2253-2257.2003 PMC152105

[b17] Linhartova I. . Meningococcal adhesion suppresses proapoptotic gene expression and promotes expression of genes supporting early embryonic and cytoprotective signaling of human endothelial cells. FEMS Microbiol Lett. 263, 109–118 (2006). 16958858 10.1111/j.1574-6968.2006.00407.x

[b18] Osicka R. . A novel “Clip-and-link” activity of repeat in toxin (RTX) proteins from Gram-negative pathogens. J. Biol. Chem. 279, 24944–24956 (2004). 15044436 10.1074/jbc.M314013200

[b19] Matyska Liskova P. . Probing the Ca^2+^-assisted π-π interaction during Ca^2+^-dependent protein folding. Soft Matter 12, 531–541 (2016). 26489523 10.1039/c5sm01796c

[b20] Kuban V., Novacek J., Bumba L. & Zidek L. NMR assignment of intrinsically disordered self-processing module of the FrpC protein of *Neisseria meningitidis*. Biomol. NMR Assign. 9, 435–440 (2015). 26138689 10.1007/s12104-015-9625-z

[b21] Sadilkova L. . Single-step affinity purification of recombinant proteins using a self-excising module from *Neisseria meningitidis* FrpC. Protein Sci. 17, 1834–1843 (2008). 18662906 10.1110/ps.035733.108 PMC2548358

[b22] Prochazkova K. . The *Neisseria meningitidis* outer membrane lipoprotein FrpD binds the RTX protein FrpC. J. Biol. Chem. 280, 3251–3258 (2005). 15525636 10.1074/jbc.M411232200

[b23] Kovacs-Simon A., Titball R. W. & Michell S. L. Lipoproteins of bacterial pathogens. Infect. Immun. 79, 548–561 (2011). 20974828 10.1128/IAI.00682-10 PMC3028857

[b24] Sviridova E. . Crystallization and preliminary crystallographic characterization of the iron-regulated outer membrane lipoprotein FrpD from *Neisseria meningitidis*. Acta Cryst. F 66, 1119–1123 (2010). 10.1107/S174430911003215X PMC2935243 20823542

[b25] Ezezika O. C. . Incompatibility with formin Cdc12p prevents human profilin from substituting for fission yeast profiling: insights from crystal structures of fission yeast profiling. J. Biol. Chem. 284, 2088–2097 (2009). 19028693 10.1074/jbc.M807073200 PMC2629104

[b26] Prlic A. . Precalculated protein structure alignments at the RCSB PDB website. Bioinformatics 26, 2983–2985 (2010). 20937596 10.1093/bioinformatics/btq572 PMC3003546

[b27] Shindyalov I. N. & Bourne P. E. Protein structure alignment by incremental combinatorial extension (CE) of the optimal path. Protein Eng. 11, 739–747 (1998). 9796821 10.1093/protein/11.9.739

[b28] Bumba L., Sviridova E., Kuta Smatanova I., Rezacova P. & Veverka V. Backbone resonance assignments of the outer membrane lipoprotein FrpD from *Neisseria meningitidis*. Biomol. NMR Assign. 8, 53–55 (2014). 23225222 10.1007/s12104-012-9451-5

[b29] Williamson M. P. Using chemical shift perturbation to characterise ligand binding. Prog. Nucl. Magn. Reson. Spectrosc. 7, 1–16 (2013). 10.1016/j.pnmrs.2013.02.001 23962882

[b30] Muller D. R. . Isotope-tagged cross-linking reagents. A new tool in mass spectrometric protein interaction analysis. Anal. Chem. 73, 1927–1934 (2001). 11354472 10.1021/ac001379a

[b31] Young M. M. . High throughput protein fold identification by using experimental constraints derived from intramolecular cross-links and mass spectrometry. Proc. Natl. Acad. Sci. USA 97, 5802–5806 (2000). 10811876 10.1073/pnas.090099097 PMC18514

[b32] Bakkes P. J., Jenewein S., Smits S. H., Holland I. B. & Schmitt L. The rate of folding dictates substrate secretion by the *Escherichia coli* hemolysin type 1 secretion system. J. Biol. Chem. 285, 40573–40580 (2010). 20971850 10.1074/jbc.M110.173658 PMC3003356

[b33] Staab J. F., Bradway S. D., Fidel P. L. & Sundstrom P. Adhesive and mammalian transglutaminase substrate properties of *Candida albicans* Hwp1. Science 283, 1535–1538 (1999). 10066176 10.1126/science.283.5407.1535

[b34] Sutherland T. C., Quattroni P., Exley R. M. & Tang C. M. Transcellular passage of *Neisseria meningitidis* across a polarized respiratory epithelium. Infect. Immun. 78, 3832–3847 (2010). 20584970 10.1128/IAI.01377-09 PMC2937448

[b35] Schaller A. . Characterization of apxIVA, a new RTX determinant of *Actinobacillus pleuropneumoniae*. Microbiology 145, 2105–2116 (1999). 10463177 10.1099/13500872-145-8-2105

[b36] Liu J. . *In vivo* induced RTX toxin ApxIVA is essential for the full virulence of *A. pleuropneumoniae*. Vet. Microbiol. 137, 282–289 (2009). 19251385 10.1016/j.vetmic.2009.01.011

[b37] Mueller U. . Facilities for macromolecular crystallography at Helmholtz-Zentrum Berlin. J. Synchrotron Radiat. 19, 442–449 (2012). 22514183 10.1107/S0909049512006395 PMC3408958

[b38] Minor W., Cymborowski M., Otwinowski Z. & Chruszcz M. HKL-3000: the integration of data reduction and structure solution – from diffraction images to an initial model in minutes. Acta Crystallogr. D Biol. Crystallogr. 62, 859866 (2006). 10.1107/S0907444906019949 16855301

[b39] Perrakis A., Morris R. & Lamzin V. S. Automated protein model building combined with iterative structure refinement. Nat. Struct. Biol. 6, 458–463 (1999). 10331874 10.1038/8263

[b40] Cowtan K. Error estimation and bias correction in phase-improvement calculations. Acta Crystallogr. D Biol. Crystallogr. 55, 1555–1567 (1999). 10489450 10.1107/s0907444999007416

[b41] Vagin A. & Teplyakov A. An approach to multi-copy search in molecular replacement. Acta Crystallogr. D Biol. Crystallogr. 56, 1622–1624 (2000). 11092928 10.1107/s0907444900013780

[b42] Murshudov G. N. . REFMAC5 for the refinement of macromolecular crystal structures. Acta Crystallogr. D Biol. Crystallogr. 67, 355–367 (2011). 21460454 10.1107/S0907444911001314 PMC3069751

[b43] Emsley P. & Cowtan K. Coot: model-building tools for molecular graphics. Acta Crystallogr. D Biol. Crystallogr. 60, 2126–2132 (2004). 15572765 10.1107/S0907444904019158

[b44] Chen V. B. . MolProbity: all-atom structure validation for macromolecular crystallography. Acta Crystallogr. D Biol. Crystallogr. 66, 12–21 (2010). 20057044 10.1107/S0907444909042073 PMC2803126

[b45] Krieger E., Koraimann G. & Vriend G. Increasing the precision of comparative models with YASARA NOVA-a self-parameterizing force field. Proteins 47, 393–402 (2002). 11948792 10.1002/prot.10104

[b46] Holm L. & Sandler C. Protein structure comparison by alignment of distance matrices. J. Mol. Biol. 233, 123–138 (1993). 8377180 10.1006/jmbi.1993.1489

[b47] Orengo C. . CATH – a hierarchic classification of protein domain structures. Structure 5, 1093–1108 (1997). 9309224 10.1016/s0969-2126(97)00260-8

[b48] Krissinel E. & Henrick K. Secondary-structure matching (SSM), a new tool for fast protein structure alignment in three dimensions. Acta Crystallogr. D Biol. Crystallogr. 60, 2256–2268 (2004). 15572779 10.1107/S0907444904026460

[b49] Blanchet C. E. . Instrumental setup for high throughput solution scattering at the X33 beamline of EMBL-Hamburg. J. Appl. Cryst. 45, 489–495 (2012).

[b50] Franke D. & Svergun D. I. DAMMIF, a program for rapid ab-initio shape determination in small-angle scattering. J. Appl. Cryst. 42, 342–346 (2009). 27630371 10.1107/S0021889809000338 PMC5023043

[b51] Kukacka Z., Rosulek M., Strohalm M., Kavan D. & Novak P. Mapping protein structural changes by quantitative cross-linking. Methods 89, 112–20 (2015). 26048481 10.1016/j.ymeth.2015.05.027

