# Supplementary material for: Structural basis of the interaction between the putative adhesion-involved and iron-regulated FrpD and FrpC proteins of Neisseria meningitidis
Source: Sci Rep. 2017 Jan 13;7:40408. doi: 10.1038/srep40408 (PMC5233953; doi:10.1038/srep40408)
Supplement: Supplementary Information [file srep40408-s1.pdf]

Supplementary Information

Structural basis of the interaction between the putative adhesion-involved and iron-regulated FrpD and FrpC proteins of *Neisseria meningitidis*

Ekaterina Sviridova, Pavlina Rezacova, Alexey Bondar, Vaclav Veverka, Petr Novak, Gundolf Schenk, Dmitri I. Svergun, Ivana Kuta Smatanova, and Ladislav Bumba

Figure S1.

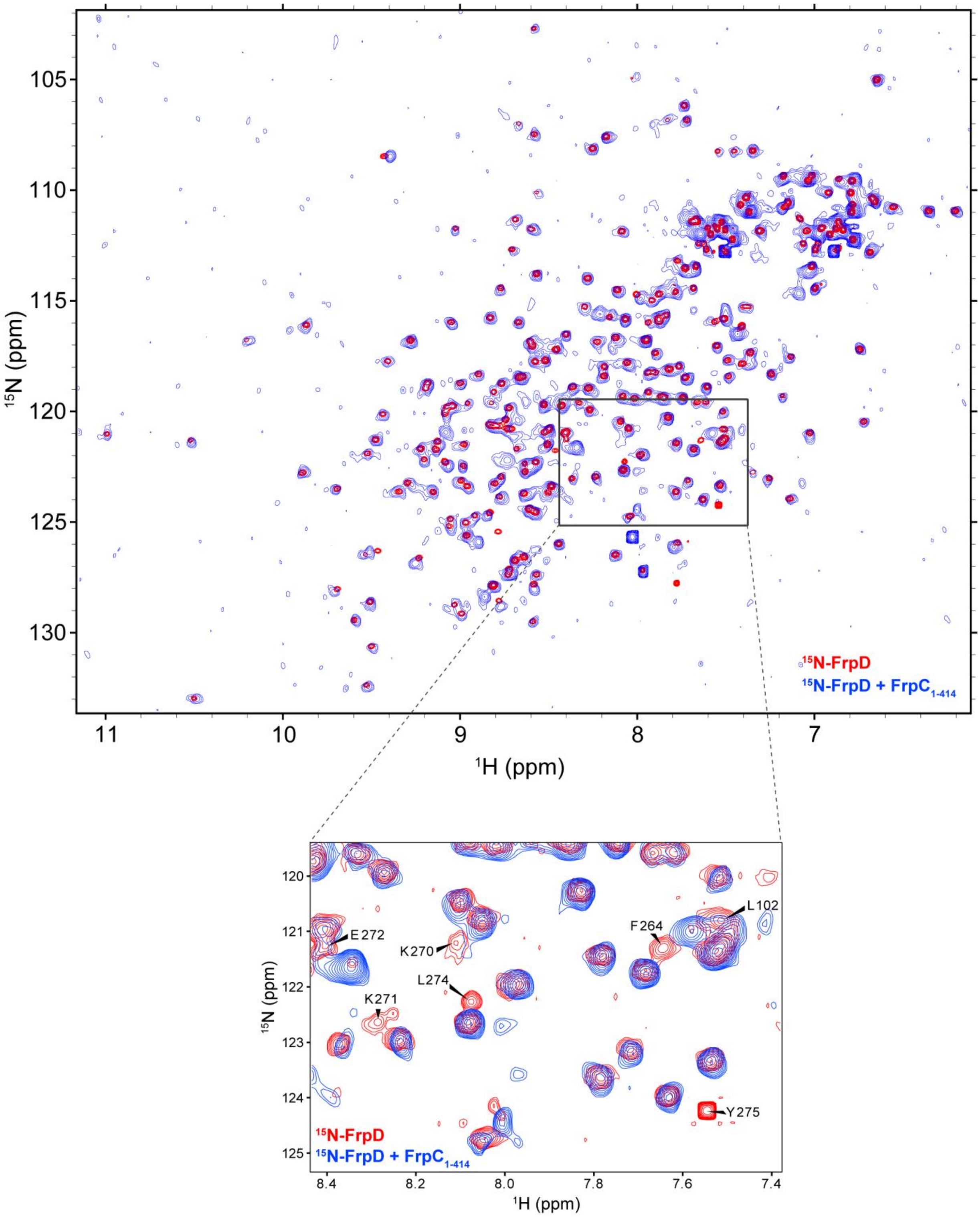

Figure S1. Overlay of the ^{15}N-^{1}H HSQC spectra of the ^{15}N-labeled FrpD in the absence (red, BMRB accession code 18779) and the presence (blue) of unlabeled FrpC$_{1-414}$ (blue). The lower panel represents the zoomed area of the spectra indicated in Figure 4C.

Figure S2.

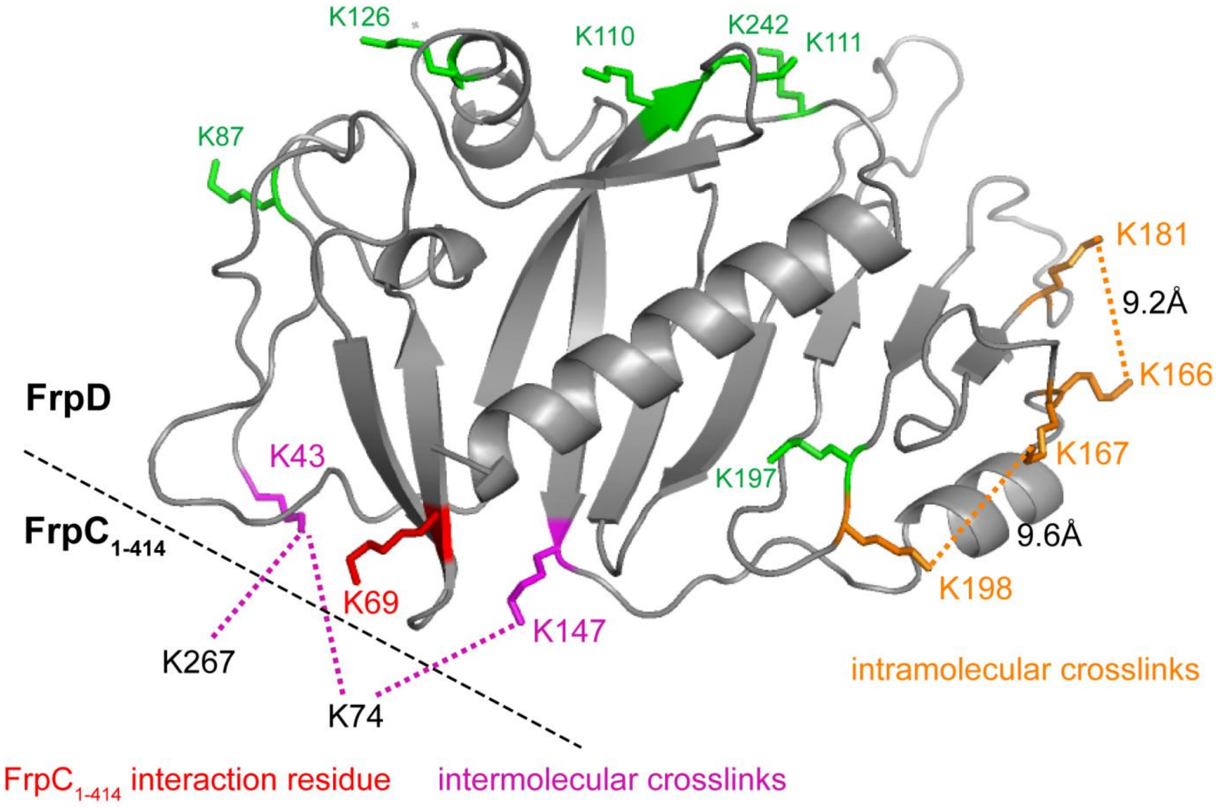

Figure S2. Schematic representation of the intramolecular crosslinks in FrpD (orange) and intermolecular crosslinks in the FrpD-FrpC$_{1-414}$ complex (magenta). The location of the lysine residues and their inter-residue distances within the FrpD structure are indicated. The K69 (red) represents the lysine residue, whose NMR signal is significantly perturbed by the FrpC$_{1-414}$ binding.

Table S1. Crystallographic statistics of the native and SeMet derivative FrpD structures.

Crystal structure	Native FrpD	SeMet FrpD
Resolution range (Å)	30.65 – 2.30	25.20 – 1.40
No. of reflections in working set	12627	44591
No. of reflections in test set	668	2388
R (%)[a]	18.3	16.8
R_{free} (%)[b]	24.9	19.3
**RMSD**		
Bond lengths (Å)	0.012	0.013
Bond angles (°)	1.27	1.43
No. of non-H atoms	1979	2217
No. of protein atoms	1849	1904
No. of PEG atoms	0	35
No. of sodium azide atoms	0	3
No. of sodium ions	0	1
No. of water molecules	130	274
Average B value (Å^2)	41.1	24.0

[a]$R=\sum_{hkl}||F_{obs}|-|F_{calc}||/\sum_{hkl}|F_{obs}|$, where F_{obs} and F_{calc} are the observed and calculated structure factors, respectively.
[b]R_{free} was monitored using 5% of the reflections chosen at random and omitted from the refinement process

Table S2. List of intermolecular (FrpD/FrpC$_{1-414}$) and intramolecular (FrpD and FrpC$_{1-414}$) crosslinks in the FrpD/FrpC$_{1-414}$ complex.

	Cross-linked residues	Cross-linked peptides	theoretical d_0-[M + H]$^+$	experimental d_0-[M + H]$^+$	Error (ppm)
FrpD/FrpC$_{1-414}$	K43/K74	1-3/62-75	2037.063	2037.069	3.0
	K43/K267	1-3/257-271	2075.057	2075.058	0.8
		1-3/257-271(M^{ox})	2091.052	2091.052	0.4
	K147/K74	107-119 (C^{CAM})/63-75	3015.434	3015.432	0.5
		107-119 (C^{CAM}, M^{ox})/62-75	3159.524	3159.523	0.3
FrpD	K166/K181	120-127/128-142	2869.427	2869.427	0.1
		120-127/128-142 (M^{ox})	2885.422	2885.422	0
	K198/K167	158-168/127-142	3311.631	3311.629	0.6
FrpC$_{1-414}$	K234/K377	231-235/373-379	1565.895	1565.895	0
	K308/K188	306-311/182-190	1728.947	1728.942	2.8
	K409/K351	406-414/345-362	3159.540	3159.534	1.8
	K405/K49	405-409/39-62 (M^{ox})	3337.763	3337.763	0.1

Table S3. Overall parameters of the SAXS experiments.

Data set	FrpD	$FrpD\text{-}FrpC_{1\text{-}414}$
**Data collection parameters**		
Instrument	EMBL X33 beamline Doris-III storage ring (DESY, Hamburg)	
Beam geometry [mm^2]	2 × 0.6	
Wavelength [nm]	0.15	
q-Range [nm^{-1}]	0.06-6.2	
Exposure time [s]	8 frames for 15 s	
Concentration range [mg/ml]	1-9	1-5
Temperature [K]	283	
**Structural parameters**		
I(0) (relative) from P(r)	16.4	50.4
Rg [Å] from P(r)	21	38
I(0) [cm^{-1}] from Guinier	16.6	50.3
Rg [Å] from Guinier	22	37
Dmax [Å]	65	130
Porod volume estimate [$Å^3$]	41000	123000
Dry volume calculated from sequence [$Å^3$][a]	32375	94200
**Molecular mass determination**		
Molecular mass MM [Da] from Porod volume (Vp x 0.6)	25000	74000
Molecular mass MM [Da] from forward scattering	13000	40000
Calculated monomeric MM [Da] from sequence	26757	73160
**Software employed**		
Data processing	ATSAS	
ab initio analysis	DAMMIF, DAMMIN, DAMAVER	

[a] The dry volume was calculated using the Peptide Property Calculator web tool available at http://www.basic.northwestern.edu/biotools/proteincalc.html.
